# Update on pulmonary hypertension

**DOI:** 10.1186/s12931-026-03635-0

**Published:** 2026-03-26

**Authors:** Horst Olschewski

**Affiliations:** Sigmund Freud Private University Vienna, Austria and Charité University Medicine, Berlin, Germany

**Keywords:** Pulmonary arterial hypertension, Group 3 PH, Chronic thromboembolic pulmonary hypertension, Targeted PAH therapy, Activin signaling inhibitor, Sotatercept, Microvascular malformations, Balloon pulmonary angioplasty

## Abstract

The hemodynamic definition of pulmonary hypertension including a mean pulmonary arterial pressure > 20 mmHg is evidence based, but the threshold of 15 mmHg for the pulmonary arterial wedge pressure, deciding between pre-and postcapillary PH, is not. Indeed, based on the most recent literature, the normal PAWP is ≤ 13.0 mmHg, raising some interesting questions. The TASK Force on treatment of pulmonary arterial hypertension (PAH) at the latest world symposium for pulmonary hypertension published a new therapeutic algorithm for PAH that looks simpler than the previous algorithm. However, this algorithm exclusively refers to high-quality evidence from pivotal studies. Sildenafil appears to be safe and effective up to 80 mg TID, combination pills including macitentan and tadalafil are available. The activin signaling inhibitor sotatercept has changed our way of thinking about PAH therapy due to unprecedented efficacy. It is highly efficacious not only in WHO FC 2–3 but also in WHO FC 4 PAH. However, according to the recent literature, the long-term effects include systemic and pulmonary microvascular malformations. The serotonin-, tyrosine kinase-, estrogen, and carboanhydrase pathways have successfully been targeted in several PH models and showed excellent safety profiles in early clinical development. However, the approval-oriented clinical studies, published in high-ranking journals, have only met their primary endpoint in case of inhaled seralutinib and inhaled MK5475. Group 3 PH comprises patients which pulmonary hypertension associated to chronic lung diseases or chronic hypoxia. However, there is an overlap between idiopathic PAH with a “lung phenotype” and Group 3 PH. In Europe, PAH targeted therapies have not been approved for group 3 PH. Treatment for COPD PH appears to be more challenging than treatment of ILD PH. However, according to a large retrospective study, sildenafil might provide beneficial effects for COPD PH patients, particularly in those with a strongly elevated PVR and a relatively well-preserved FEV_1_. For chronic thromboembolic pulmonary disease, pulmonary endarterectomy is therapy of choice. If this is not feasible, balloon pulmonary angioplasty is superior to medical therapy with riociguat. However, among medical therapies, riociguat still appears to be the most reliable medication that is globally approved for CTEPH.

## Background

Pulmonary hypertension affects about 1% of the world population and is associated with high morbidity and mortality, even if it is only mild. For a small subset of these diseases, targeted therapies are available that have been shown to be efficacious and safe. During the last World Symposium on Pulmonary Hypertension in Barcelona, 2024, updated diagnostic and therapeutic algorithms were developed and there is new literature on therapy of PAH, group3 PH and CTEPH.

This update refers to the published literature of April 2024 to May 2025 from clinical high-impact journals and the ATS conference 2025, San Diego. The chapters comprise hemodynamic definitions of PH, and therapy, focusing on pulmonary arterial hypertension (group 1, PAH), pulmonary hypertension associated with chronic lung disease (group 3 PH), and chronic thromboembolic pulmonary hypertension (group 4 PH, CTEPH).

### Hemodynamic definition of pulmonary hypertension

About 1% of the world population is affected by pulmonary hypertension [1] and this is associated with a significantly increased mortality, even if the pressure elevation is only mild [2–4]. Current guidelines are fully aware of these facts, however, targeted therapies for pulmonary hypertension are only available for a small subset of these patients [5]. This chapter will focus on the new definition of pulmonary hypertension, with a view on the newest evidence for the role of pulmonary arterial wedge pressure (PAWP) and exercise pulmonary hypertension.

#### Definition of PH

The Task Force on Definition and Classification of PH at the 7th World Symposium on Pulmonary Hypertension (7thWSPH) in Barcelona, 2024, published their proceedings, representing the current state of the art [6].

As depicted in Table 1, pulmonary hypertension is defined by a mean pulmonary arterial pressure above 20 mmHg at rest. This applies for lowland but not highland conditions, where the normal pulmonary arterial pressure may be higher. Precapillary PH is defined by a maximum pulmonary arterial wedge pressure of 15 mmHg and a pulmonary arterial resistance (PVR) above 2 Wood units (WU), while postcapillary PH is defined by a PAWP above 15 mmHg. If postcapillary PH is associated with a PVR < 2 WU, it is classified as isolated postcapillary PH (ipcPH), if it is associated with a PVR > 2 WU, it is classified as combined pre-and postcapillary PH (cpcPH). This is similar to the previous definition with the exception that the PVR threshold changed from 3 to 2 WU. 

The definition for exercise PH has not changed since the 6thWSPH, 2019, and refers to a mean pulmonary arterial pressure/cardiac output (mPAP/CO) slope > 3 WU.

**Table 1:** Haemodynamic criteria of pulmonary hypertension (PH), from Kovacs et al. ERJ 2024 [7]

	Haemodynamic characteristics
PH	mPAP > 20 mmHg
Pre-capillary PH	mPAP > 20 mmHg
	PAWP ≤ 15 mmHg
	PVR > 2WU
Isolated post-capillary PH (ipcPH)	mPAP > 20 mmHg
	PAWP > 15 mmHg
	PVR ≤ 2 WU
Combined post- and pre-capillary PH (cpcPH)	mPAP >20 mmHg
	PAWP >15 mmHg
	PVR >2 WU
Exercise PH	mPAP/CO slope > 3 mmHg/L/min between rest and exercise

*mPAP* mean pulmonary arterial pressure, *PAWP* Pulmonary arterial wedge pressure, *PVR* Pulmonary casvular resistance, *WU* Wood units, *CO* Cardiac output

#### Exercise PH

**Fig. 1:**
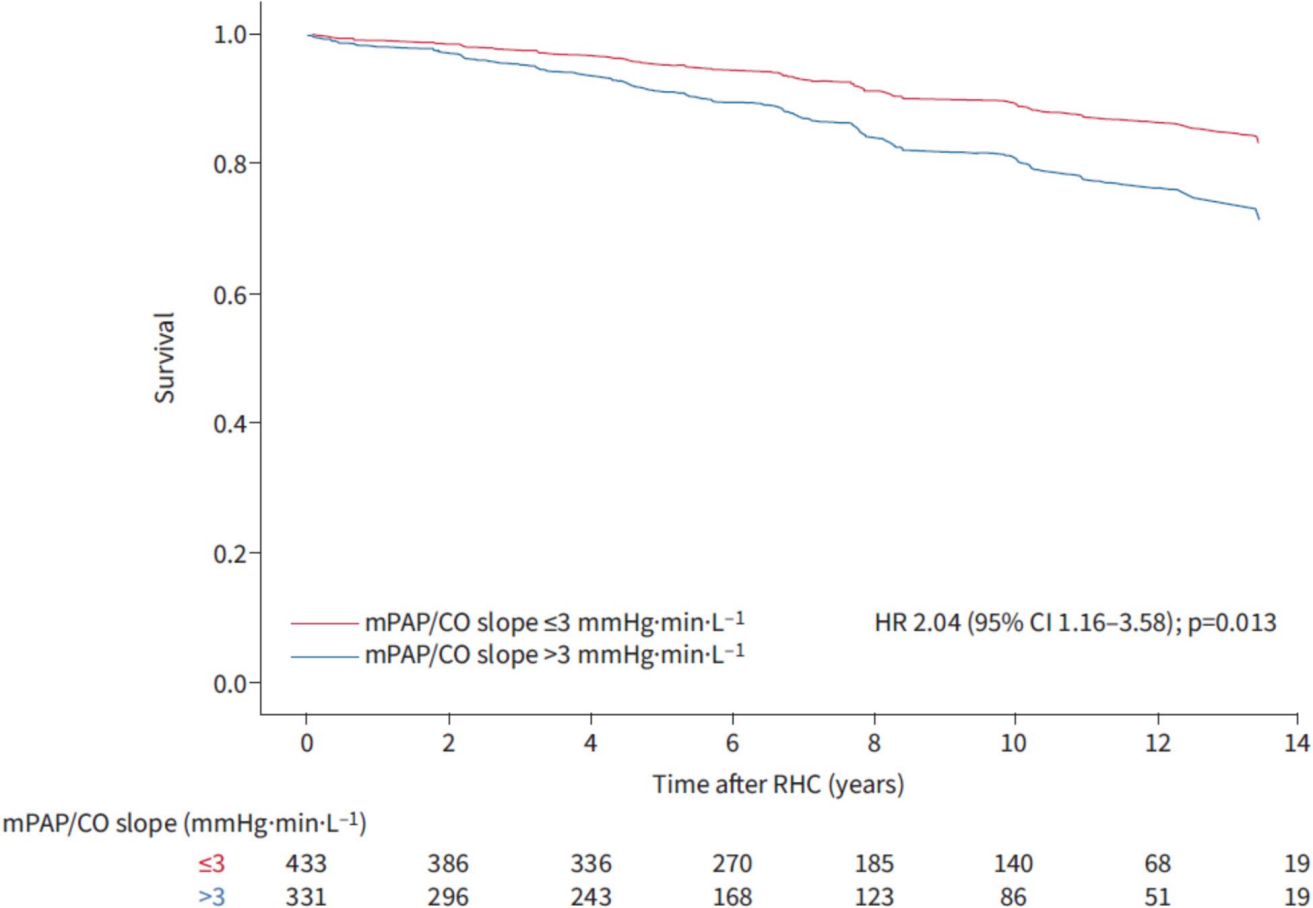
Kaplan Meier curve for overall survival, from Kovacs et al. ERJ 2024 [8].

There is new evidence for the prognostic importance of exercise PH. The PEX-NET consortium, supported by the European Respiratory Society, analyzed 724 patients who had been enrolled in their international registry from 23 European and American PH clinics. All patients had a resting mPAP < 25 mmHg, and had undergone RHC measurements at rest and maximum exercise in the same body position [8]. The primary endpoint of this retrospective analysis was all-cause mortality. After correction for age, sex, hemoglobin and resting hemodynamics, three independent predictors of mortality remained: the maximum achieved CO (the higher the better), the maximum transpulmonary gradient (the lower the better) and the mPAP/CO slope, again the lower the better. The hazard ratio for the mPAP/CO slope was 2.0 [95% confidence interval 1.2—3.6], and the respective Kaplan Meier curve for all-cause mortality over the next 14 years was impressive (Fig. 1).

This result supports and confirms the actual definition of exercise PH as mPAP/CO slope > 3 WU.

### Pulmonary arterial wedge pressure (PAWP)

A recent meta-analysis, the largest that has ever been conducted, including 960 healthy controls with right heart catheterization (RHC), showed that the normal PAWP is about 10 mmHg, and the double standard deviation is about 3 mmHg, resulting in an upper limit of normal of 13 mmHg [9]. This is a robust result, even when potential covariates like age, BMI and sex are considered, or when only aggregated or only individual data are considered. However, this result causes kind of a gap between the upper limit of a normal PAWP and the PAWP threshold for postcapillary PH which is > 15 mmHg and which was used as exclusion criterium in all pivotal studies for PAH and CTEPH. Therefore, it was an important question, if in patients with a PAWP between 13 and 15, these medications were as good as in patients with a PAWP below 13 mmHg. This was addressed in an analysis of 4337 individual patient data from all the randomized placebo-controlled trials, submitted to the FDA, with two endpoints, the change in 6 min walking distance (6mwd) and time to clinical worsening. As main result, the beneficial drug effects were independent of PAWP. This was also independent of the tested drug [10].

### This does not mean that PAWP does not matter anymore!

The PASSION trial included patients with cpcPH, who were randomized to the phosphodiesterase 5 inhibitor (PDE5i) tadalafil or placebo [11]. Their hemodynamics were characterized by mPAP = 42 mmHg and PAWP = 20 mmHg. Unfortunately, the study was prematurely terminated due to missing drug supply, however, it had already enrolled 125 patients that could be formally analyzed. The primary endpoint, the time to hospitalization or death showed no clear trends, however, all-course mortality, as part of the primary endpoint, was in favor of placebo! This suggests that cpcPH should not be treated with a PDE5i, and this may be generalized to all current PAH drugs, because there is not a single high-quality study showing evidence for beneficial effects of any PAH drug in patients with PAWP > 15 mmHg.

## Conclusions for Chapter 1

PH is defined by a mean pulmonary arterial pressure > 20 mmHg, exercise PH by a mPAP/CO slope > 3 WU, both based on strong evidence. However, the thresholds for pre-vs. postcapillary PH (≤ 15 vs. > 15 mmHg) are still arbitrary and non-evidence based. There is no high-quality evidence for PAH drugs in patients with PAWP > 15 mmHg. 

### PAH therapy

**Fig. 2:**
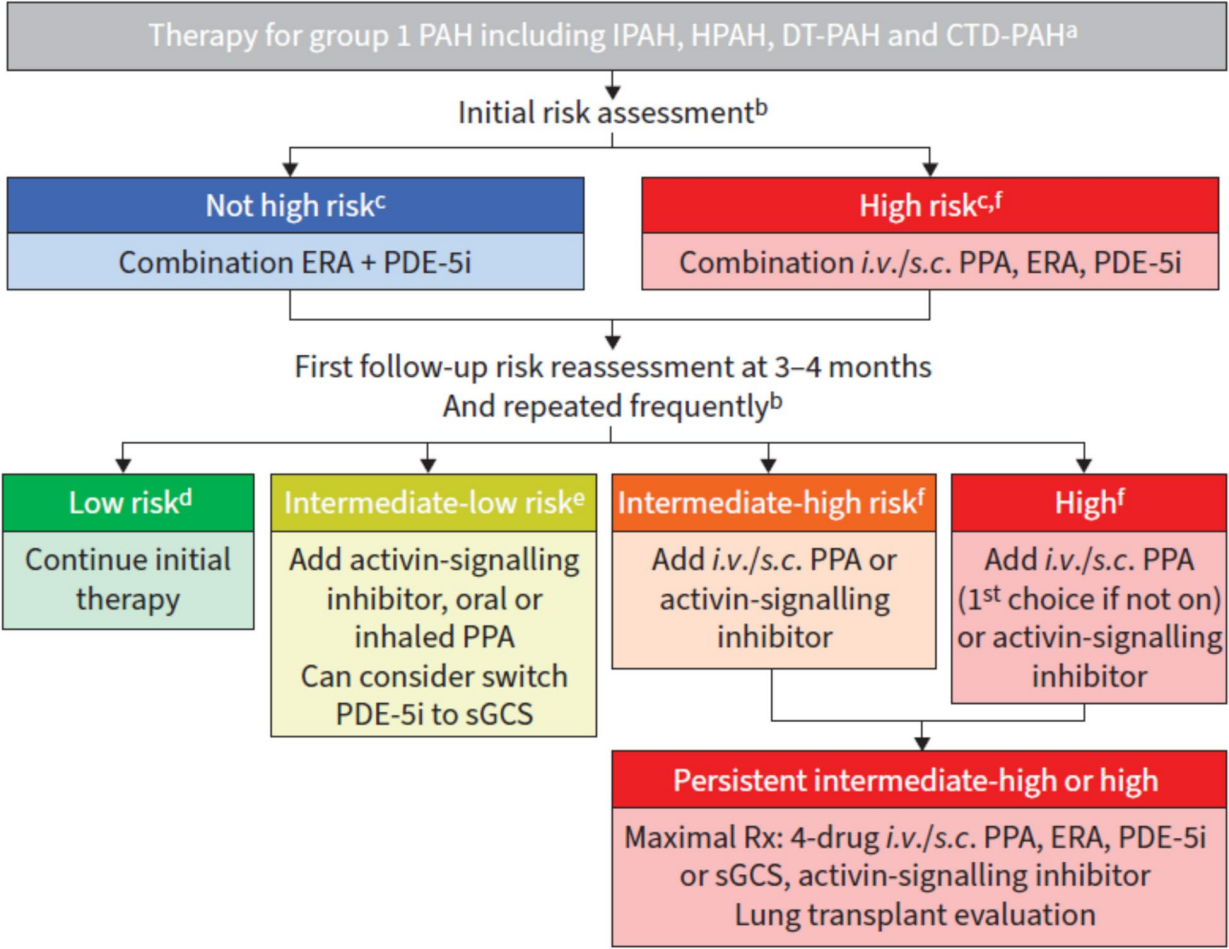
WSPH therapy algorithm, from Chin et al. ERJ 2024 [12].

The TASK Force on treatment of pulmonary arterial hypertension (PAH) at 7thWSPH published a new therapeutic algorithm for PAH (Fig. 2) that looks a lot simpler than the previous algorithm [12]. However, this algorithm refers only to high-quality evidence from pivotal studies, as indicated in the first line of the algorithm.

Superscripts a. The treatment algorithm is intended for patients with confirmed group 1 PAH (phenotypically clear-cut, including mPAP ≥ 25 mmHg and PVR > 3 Wood Units and no significant response on acute vasoreactivity testing). b. *Risk assessment* should be performed at baseline, within 3–4 months and periodically thereafter, and using FC, 6MWD and natriuretic peptides as a part of a validated risk calculator. Haemodynamics, RV imaging and other measures should be used to supplement risk assessment. c. *Initial triple therapy* with an *i.v*./*s.c*. PPA is recommended in high-risk patients and may be considered in non-high risk with severe haemodynamics and/or poor RV function. d. Most *low-risk patients* at follow-up should continue initial therapy. e. Clinical trials with oral and inhaled treprostinil included *only patients on monotherapy*, while studies of selexipag and sotarcept included patients on combination therapy. f. *Transplant referral* should be considered for select high-risk patients at diagnosis, and for intermediate-high and high.

This means, the algorithm excludes portopulmonary hypertension, HIV-associated PH, PVOD, and schistosomiasis-associated PH. This also indicates that it excludes those with PAH and mPAP < 25 mmHg or PVR < 3 WU and all the old and multimorbid patients that would not have qualified for the pivotal studies.

According to the algorithm, these “typical patients”, mostly go on an oral combination therapy with an endothelin receptor antagonist (ERA) and a PDE5i, and then undergo re-evaluation of their mortality risk. After that, they will face therapy escalation, except they are in the green zone, corresponding to a low mortality risk. Escalation can consist of adding an activin signaling inhibitor (ASI), switching from a PDE5i to riociguat, adding an inhaled, subcutaneous or intravenous prostacyclin, or even considering lung transplantation. This suggests that this algorithm is relevant for patients who principally qualify for lung transplantation, based on age and comorbidities.

### Re-evaluation after 3–4 months

Therapy decisions are based on regular re-evaluations, the first of which should be done 3–4 months after starting a patient on a new PAH therapy. The algorithm uses the categories low, intermediate-low, intermediate high and high risk, which relate to the COMPERA2.0 risk strata [13].

The Task Force on Risk Stratification and Therapy Goals at the 7thWSPH has discussed the current evidence for risk prediction in PAH and provided a graphical abstract.

**Fig. 3:**
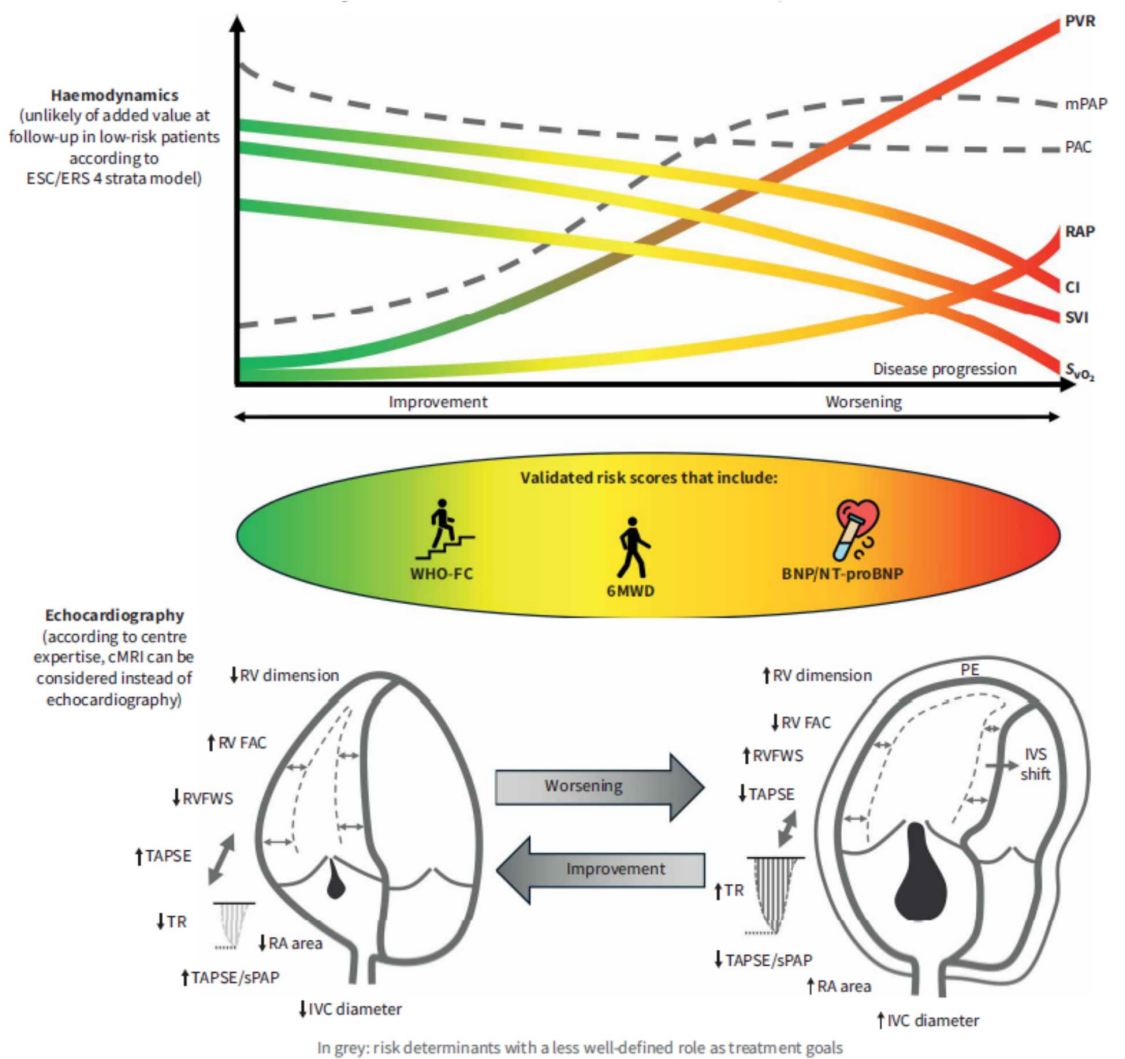
WSPH Task Force graphical abstract, from Dardi et al. ERJ 2024 [6].

The upper graph in Fig. 3 shows the development of the hemodynamics, as assessed by RHC, where even today the diagnosis is mostly made in the last third of the time axis. In this phase, the natural course of the disease is characterized by a rising PVR, a decreasing cardiac output, central venous oxygen saturation and stroke volume, and a more or less stable mPAP. In the middle graph, you see the most important measures for risk stratification in all the established risk scores, WHO-Functional Class (WHO FC), 6 min walking distance (6mwd) and B-type natriuretic peptide (BNP). However, the Task Force provided a critical discussion of these measures, stating that they all strongly depend on age and comorbidities [6]. And at the bottom graph, non-invasive measures of right ventricular function are shown, which are mostly derived from echocardiography but can be derived in a similar way from cardiac MRI. The intention of the Task Force was to suggest to consider the whole picture of a patient, before proceeding to important therapy decisions on PAH therapy.

There was a recent refinement of the 4-strata to a 6-strata risk score by means of including the hemodynamics from RHC. The analysis was based on 1240 patients from the French PH registry [14]. In the patients in the intermediate risk groups, a stroke volume index > 37 mL/m^2^ or a central venous oxygen saturation > 65%, adds significant prognostic information.

### The four pathways of PAH drugs

All our approved drugs are based on 4 different molecular pathways (Table 2) and it seems that all combinations of drugs are beneficial, provided they are addressing different pathways. The endothelin pathway includes dual blockers of ETA and ETB receptors and selective ETA blockers, the NO pathway includes PDE5i and stimulators/activators of soluble guanylate cyclase (sGCs), the prostacyclin pathway includes activators of the prostacyclin receptor (PRA) consisting of prostanoids (PGI_2_ and synthetic analogues) or non-prostanoid PRAs, and the activin signaling pathway, consists of an activin signaling inhibitor (ASI), which is applied subcutaneously.

**Table 2:** All approved PAH therapies are based on 4 pathways, the endothelin (ERA), the nitric oxide (NO), the prostacyclin (PRA), and the activin signaling pathway (ASI)

	Approved dose	Pathway
Ambrisentan	5–10 mg OD	ERA
Macitentan	10 mg OD	ERA
Bosentan	125 mg BID	ERA
Tadalafil	20–40 mg OD	NO
Sildenafil	20 mg TID	NO
Riociguat	1–2.5 mg TID	NO
Epoprostenol	iv	PRA
Treprostinil	sc., iv, (inhaled,FDA)	PRA
Iloprost	inhaled	PRA
Selexipag	200–1600 mg BID	PRA
Sotatercept	sc ≤ 0.7 mg/kg/3wk	ASI

Before describing the exciting effects of ASI, it is important to acknowledge two recent publications, about combination pills for PAH and the sildenafil dose.

### Macitentan/Tadalafil combination pills

There are many arguments for combination pills containing macitentan and tadalafil. These drugs are efficacious, affect different molecular pathways, have a similar half-life and very few drug-drug interactions and there is evidence for beneficial combination effects from high-quality clinical trials.

An actual phase 1 trial has shown quite perfect pharmacologic properties of macitentan + tadalafil combination pills [15] and such combination pills in a dose of 10 + 20 mg and 10 + 40 mg have been provided by pharmaceutical industry.

### Sildenafil dose

Previously, based on the STARTS-2 trial, which showed excess mortality of children and young adults on higher doses of sildenafil [16], there was a warning towards use of higher doses than 20 mg TID, and the FDA requested a prospective safety study, which has been published recently. This doubleblind prospective trial randomized patients to 5 mg, 20, or 80 mg TID long-term. A total of 385 patients was analyzed. The primary endpoints were time to clinical worsening and change in 6mwd. The study was prematurely terminated because interim data strongly favored the higher doses of sildenafil. The highest dose (80 mg TID) achieved the best results concerning time to clinical worsening and was equivalent to 20 mg TID in the change of 6mwd, while the 5 mg TID dose was significantly inferior [17]. This suggests that the STARTS-2 trial had been biased in favor of the lower doses and that the high dose, up to 80 mg TID may be effective and safe.

## Conclusions for Chapter 2

PAH therapy relies on four pathologic pathways, the endothelin-, NO-, prostacyclin, and activin signalling pathway. Sildenafil appears to be safe and effective up to 80 mg TID, combination pills including macitentan and tadalafil are available.

### Activin signaling inhibitors

This chapter continues with PAH therapies, but due to the novelty of the approach, it was given a chapter of its own. The activin signaling inhibition by sotatercept has changed our way of thinking about PAH therapy. However, in addition to favorable pulmonary hemodynamic changes, according to the recent literature, this new drug is also associated with systemic and pulmonary microvascular malformations.

Sotatercept is the first approved ASI. It is applied subcutaneously every 3 weeks in a dose up to 0.7 mg/kg. The Phase 2 study, PULSAR [18] and the pivotal Phase 3 study, STELLAR [19] showed an excellent safety profile and remarkable efficacy concerning clinical and hemodynamic benefits [20]. However, approval was restricted to WHO FC II and III, because patients in WHO FC IV had been excluded from the trials. The recently published ZENITH study has closed this gap. It included patients in WHO FC III and IV despite dual or triple combination therapy [21]. The primary endpoint was a combination of hospitalization for > 24 h for PAH deterioration, all-cause mortality or lung transplantation after 24 weeks. The study was prematurely terminated by the Steering Committee because there were strong signs for superiority of drug vs. placebo. A total of 272 patients had been randomized and the hazard ratio for the primary endpoint was 0.24 [95% CI 0.13–0.43] in favor of the drug. The placebo group performed quite poorly with an event-free survival after 1 yr of only about 50%. The patients were mainly female, and a little older than in PULSAR and STELLAR (54 yr), 28% were associated with scleroderma, and 26% were in WHO FC IV. The baseline 6mwd was only 270 m and nt-pro BNP was severely elevated to 3150 pg/mL. Statistical evaluation of the secondary endpoints was hampered due to the premature termination of the study, however, it showed a remarkable decrease of overall mortality (HR 0.42 [CI0.17–1.07] and transplant free survival (HR 0.34 [0.15–0.78], a decrease in mPAP by 21 mmHg and an increase in 6mwd by 63 m, when the Hodges Lehman estimate is considered. Such an improvement is unprecedented by any study applying a PAH drug on top of a strong PAH therapy. But ZENITH was a 24 week study, like PULSAR and STELLAR.

### Long-term effects of sotatercept

Patients who finished the placebo-controlled sotatercept studies were eligible for the long-term safety study SOTERIA. The 1 yr results have just been published [22] and found that the beneficial effects on WHO FC and physical capacity were maintained over the next year and that placebo patients completely catched up with the sotatercept patients after about half a year – which is different from the prostacyclin experience, where the placebo patients never catched up. In terms of safety, there were 30% serious adverse events, however, less than 3% were considered to be drug-related. There were adverse events of special interest (AESI), teleangiectasia and nose bleeding. Both occurred in around 20% of patients within the first year and there were severe bleeding events in about 5%, however, again not considered treatment-related by the investigators. At the ATS conference in San Francisco, in May 2025, Iona Preston, the first author of SOTERIA, gave an update of the SOTERIA data. She said that the AESIs continue to develop with about 11% per year in the second and third year of therapy and that severe bleeding was also seen with an incidence of about 5% (Preston, I. oral presentation, ATS conference, 2025). This suggests that sotatercept can cause dysfunctional vasculogenesis and bleeding in the systemic vessels.

### Dysfunctional vasculogenesis in the pulmonary vessels, also?

Recent findings suggest that sotatercept also may cause dysfunctional vasculogenesis in the pulmonary vessels resulting in severe hypoxemia. Two very well documented case reports, one from Hannover and one from Paris, describe patients who developed pulmonary capillary dilatations and severe hypoxemia after about a year of sotatercept treatment, partially reversible after sotatercept cessation [23]. The authors speculate, this might be due to an interaction of sotatercept with bone morphogenetic protein (BMP) 9 and BMP 10, which are known for their important functions in vascular homeostasis. However, this is speculative and more evidence is needed.

### Unprecedented efficacy and consequences for concomitant medication

Patients with an excellent response to sotatercept may ask, if they really need to continue with their intravenous or subcutaneous prostacyclin therapy. The Hannover group has just recently published their experience with 27 patients on sc. treprostinil, who received additional sotatercept. Out of these, 10 patients had favorable effects of sotatercept and were completely weaned from treprostinil. The hemodynamic changes are quite remarkable.

As shown in Table 3, before sotatercept add-on, patients had very severe pulmonary hypertension despite treprostinil. This improved very much after sotatercept, including a decrease of mPAP by 21 mmHg. Weaning of treprostinil caused another decrease of mPAP by 6 mmHg and an increase of 6mwd by another 40 m. This suggests that these patients did not only profit from sotatercept addition but also from treprostinil weaning. However, as the authors state, more evidence is needed before recommendations on the right prostacyclin strategy in patients on sotatercept can be made.

**Table 3:** Hemodynamic effects of sotatercept (sota) add-on and treprostinil (tre) weaning in 10 patients with favorable sotatercept effects. Data from Olsson et al. 2025 [24]

	Before sota on tre	6 mon on sota + tre	0-7mon after tre weaning
mPAP, mmHg	64	43	37
PVR, WU	14.1	7.8	8.3
CI, I/min/m^2^	2.5	2.6	2.4
BNP, pg/ml	1138	200	186
6 mwd, m	328	505	546

Mean values of 10 patients weaned from treprostinil

## Conclusions for chapter 3

The activin signaling inhibitor sotatercept is highly efficacious not only in WHO FC 2–3 but also in WHO FC 4 PAH. Treatment emergent effects of special interest are systemic and pulmonary microvascular malformations, developing gradually over months and years.

### Other novel therapeutic pathways

The serotonin-, tyrosine kinase-, estrogen, and carboanhydrase pathways were successfully applied in several PH models and showed excellent safety profiles in early clinical development.

As shown in Table 4, several high-quality randomized controlled trials have been published in high-ranking journals. Out of these 6 studies, 4 did not meet their primary endpoint, i.e. they were negative, although there were very good data from preclinical studies and early phase clinical data. This applies to rodatristat [25], inhaled imatinib [26], anastrozole [27], and acetazolamide [28]. Only 2 of these studies met their primary endpoints, inhaled seralutinib [29] and inhaled MK-5475 [30], however, even in these successful studies, the change in 6mwd was not significant (NS). Nevertheless, these two inhaled substances have entered phase 3 studies, seralutinib for PAH, and MK-5475 for PAH and COPD-PH, which is, by the way, an ambitious indication.

**Table 4:** New PAH therapeutic approaches working on other than the 4 established therapeutic pathways

Acronym	Substance	Pathway	Outcome	Journal
Elevate-2	Rodatristate	Serotinin antagonist	Negtive	Lancet Respir Med
	Inhaled imatinib	Tyrosin Kinase Inhibitor	Negative	Pulm Circ
PHANTOM	Anastrozole	Estrogen antagonist	Negative	AJRCCM
-	Azetazolamid	Carboanhydrase inhibitor	Negative	Pulmonology
TORREY	Inhaled seralutinib	Tyrosin Kinase Inhibitor	PVR significant; 6mwd NS	Lancet Respir Med
INSIGNIA-PAH	Inhaled MK-5475	Soluble guanylyl cyclase stimulator	PVR significant; 6mwd NS	ERJ

## Conclusion for Chapter 4

A couple of clinical studies, targeting other therapeutic pathways than activin signaling inhibition, have been published in high-ranking journals. They may not have attained the attention they deserve.

### Group 3 PH

Group 3 PH comprises patients with pulmonary hypertension associated to chronic lung diseases or chronic hypoxia or combinations of these. There is an overlap between idiopathic PAH with a “lung phenotype” and Group 3 PH. In Europe, PAH targeted therapies have not been approved for group 3 PH. The 7thWSPH Task Force on Definition and Classification changed the classification of Group 3 PH subgroups using underlying diseases like “COPD” or “interstitial lung disease”, instead of terms like “obstruction” and “restriction” [7]. This chapter will focus on COPD and ILD.

It is still state-of-the-art, that therapy with PAH drugs is not recommended in Group 3 PH [5]. Exceptions may be made, if PH is very severe, i.e. PVR > 5 WU, and the patient is being treated by a PH expert center. ILD with severe PH is considered as a possible indication for PDE5i, but only in severe PH and not in non-severe PH. Inhaled treprostinil (iTre) may be considered, based on the INCREASE study, that led to the approval for iTre in ILD PH in the US, but not in Europe [31]. INCREASE had shown that ILD PH patients, randomized to iTre vs. placebo, had significant benefits in change of 6mwd (primary endpoint) and change in nt-pro BNP and clinical worsening (secondary endpoints).

The same study protocol was recently applied to COPD PH and the acronym of the study was PERFECT, while the results were not perfect at all [32]. The study was early terminated due to safety concerns. The primary endpoint showed no effects but serious adverse effects—and deaths were more common with drug as with placebo (n = 17 vs. 6 and 5 vs. 1). This suggests that it makes a big difference if we talking about ILD PH or COPD PH.

### Any positive results in COPD PH?

**Fig. 4:**
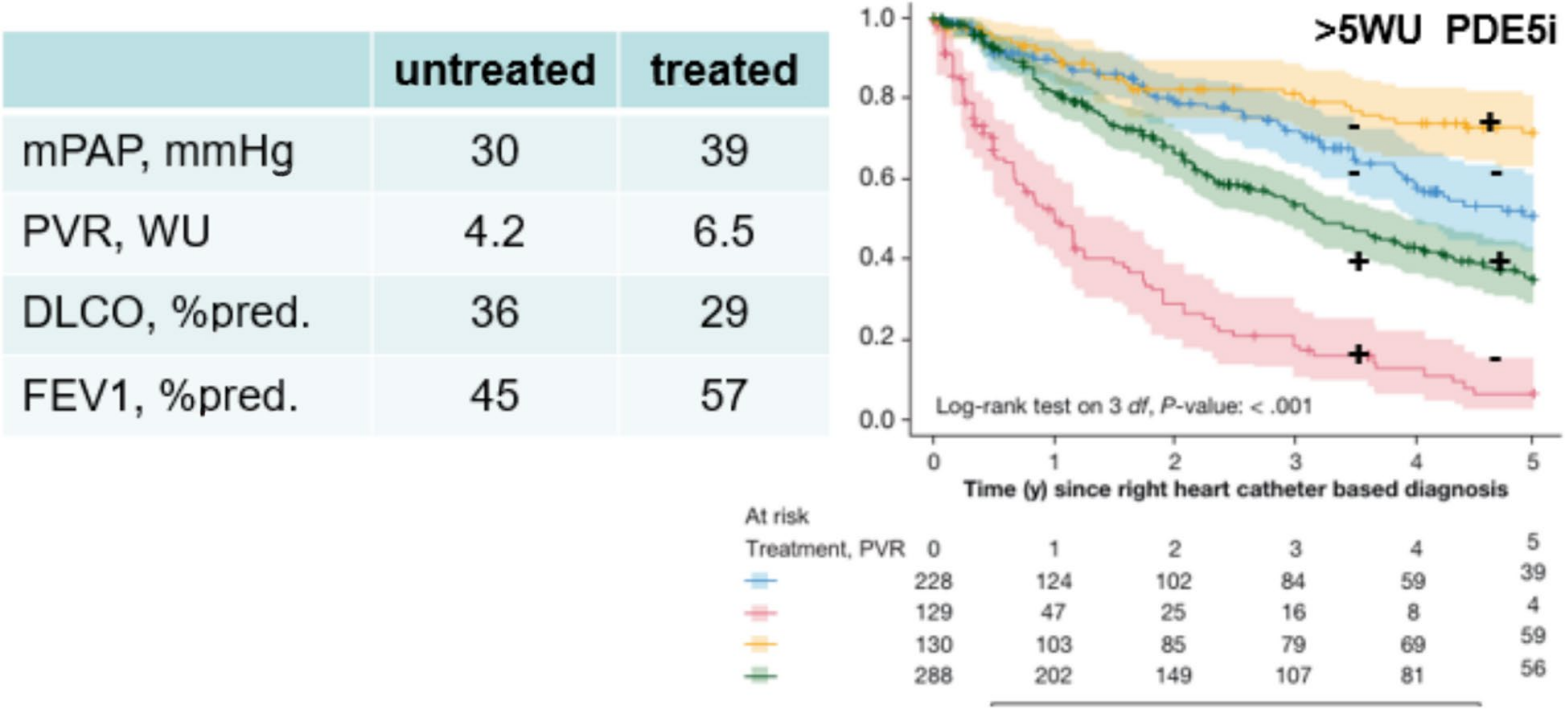
Kaplan Meier plot of overall survival from Tello et al. Chest 2024 [33].

The GO DEEP meta-registry provides evidence that COPD PH patients may profit from PAH therapy in terms of mortality (*p* < 0.001) as impressively shown in the Kaplan Meier plot (Fig. 4).

The 836 analyzed patients were on average 66 yr old, had an FEV_1_ of 51%, an mPAP of 35 mmHg and a PVR of 5 WU [33]. If these patients are stratified by PVR below and above 5 WU (Fig. 4), the beneficial effects of the PDE5i becomes even more obvious than in the overall cohort. In patients with PVR > 5 WU, the treated vs. untreated groups (green vs. red plot) separate from the beginning, while in the patients with PVR < 5 WU, the treated vs. untreated groups (orange vs. blue plots) start separating only after 4–5 yr.

However, this was a retrospective study. Therefore, it is worthwhile to have a closer look on the features of the treated vs. untreated patients. The treated patients had significantly higher mPAP and PVR and lower DLCO (39 vs. 30 mmHg; 6.5 vs. 4.2 WU; 29 vs. 36% predicted, respectively), suggesting a worse survival than the untreated group. This makes their survival benefit on PDE5i even more impressive! But they also had a better-preserved airway function (FEV_1_ 57 vs. 45% predicted). This suggests that a majority of these patients corresponded to the “vascular phenotype” of COPD, and that particularly these patients had a mortality benefit of PAH therapy.

## Conclusions for Chapter 5

Treatment for COPD PH appears to be more challenging than treatment of ILD PH. However, according to a large retrospective study, sildenafil might have beneficial effects on COPD PH patients, provided they have a strongly elevated PVR and a relatively well-preserved FEV_1_.

### Chronic thromboembolic pulmonary hypertension (CTEPH)

**Fig. 5:**
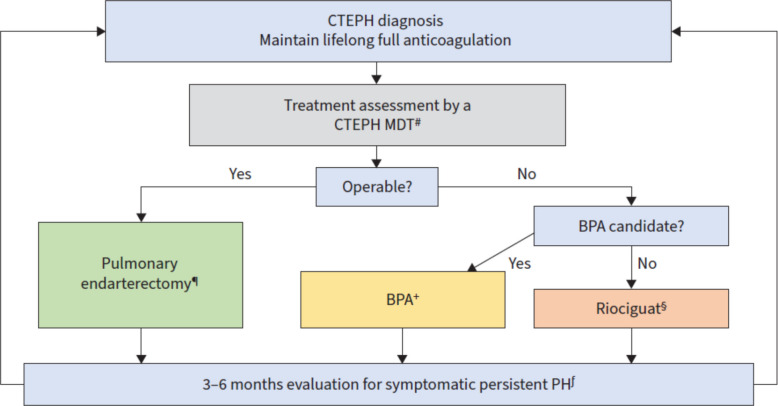
WSPH therapy algorithm for CTEPH from Kim et al. ERJ 2024 [34].

Chronic thromboembolic pulmonary disease can be associated with pulmonary hypertension (CTEPH) or without pulmonary hypertension. Even without pulmonary hypertension, it may cause severe dyspnea due to ventilation/perfusion mismatch and wasted ventilation. It is state of the art, that patients after diagnosis of chronic thromboembolic disease receive lifelong full anticoagulation and assessment for operability. This is shown in the actual treatment algorithm of the 7thWSPH Task Force (Fig. 5) [34]. If patients can be operated by means of pulmonary endarterectomy, they should actually be operated. If not, balloon pulmonary angioplasty (BPA) should be performed, and if not possible, medical therapy is indicated.

For medical therapy, there is a worldwide approval for riociguat and a European approval for iv. or sc. treprostinil. As compared to the previous algorithm, the order of medical treatment and BPA has changed and BPA comes first. Among the arguments for this change, riociguat had less beneficial hemodynamic effects as compared to BPA, and the alternative drug, treprostinil, causes local and sometimes peripheral pain.

The comparison between BPA and riociguat was made in the RACE trial. RACE randomized 100 non-operable CTEPH patients to primary BPA vs. primary riociguat treatment [35]. A recent post-hoc analysis of the hemodynamic outcomes after 1 yr [36] shows that the effects on PVR, mPAP, pulmonary arterial compliance, and RV function were all superior in BPA as compared to riociguat. But what if riociguat is just not the best medication? Because of this question, randomized controlled trials with alternative drugs are very much appreciated.

### Macitentan and selexipag for non-operable CTEPH

The SELECT trial compared selexipag, a prostanoid receptor agonist, with placebo, in non-operable CTEPH. The study was sufficiently powered, well designed, and conducted, but discontinued for futility, after it showed no treatment effect on its primary endpoint, the change in PVR [37]. The MACITEPH trial, comparing macitentan, 75 mg OD, with placebo, was also terminated for futility, but details remain unpublished at this time, except for the announcements from ClinicalTrials.gov and the sponsor.

## Conclusions for Chapter 6

In inoperable CTEPH, balloon pulmonary angioplasty is the preferred therapy option vs. medical therapy with riociguat. However, among medical therapies, riociguat appears to be the most reliable medication that has been globally approved for CTEPH. Currently there is no evidence for beneficial effects of medical combination therapies in CTEPH.

## Data Availability

Published literature available from pubmed and ATS conference, 2025.
